# Fertility desires, unmet need for family planning, and unwanted pregnancies among HIV-infected women in care in Kinshasa, DR Congo

**DOI:** 10.11604/pamj.2015.20.235.5859

**Published:** 2015-03-12

**Authors:** Marcel Yotebieng, Alison Norris, Jean Lambert Chalachala, Yori Matumona, Habib Omari Ramadhani, Frieda Behets

**Affiliations:** 1 Ohio State University, College of Public Health, Division of Epidemiology, Columbus, OH; 2 University of North Carolina at Chapel Hill, Department of Epidemiology, Chapel Hill, NC; 3 University of Kinshasa, School of Public Health, Kinshasa, DR Congo; 4 University of North Carolina at Chapel Hill, School of Medicine, Chapel Hill, NC

**Keywords:** HIV, unwanted pregnancy, family planning, contraceptives, DR Congo

## Abstract

**Introduction:**

We assessed the fertility desires, utilization of family planning (FP) methods, and incidence of pregnancies among HIV-infected women receiving care in an HIV clinic with an onsite FP services in Kinshasa, Democratic Republic of Congo.

**Methods:**

Between November 2011 and May 2012, all HIV-infected women who attended a routine visit at the clinic were interviewed about their fertility desires and utilization of contraceptive methods using a structured questionnaire. Routine follow-up visit data were used to identify pregnancies recorded between the interview and June 2013.

**Results:**

Overall, of the 699 HIV-infected women interviewed. 249 (35.7%) reported not wanting another child. Of the 499 (72.2%) participants who were sexually active at the time of interview, 177 (35.5%) were using an effective contraceptive method, including 70 (14.0%) women who reported using condoms consistently and 104 (20.8%) who were using injectable contraception. Overall, 88 (17.6%) sexually active participants who did not want another child were not using an effective FP method, and thus are considered to have had unmet need. During the median follow-up time of 22.2 (IQR: 20.2, 23.6) months, among all women interviewed, 96 (14.1%) became newly pregnant [pregnancy rate 9.3 (95%CI: 7.6, 11.4) per 100 women-years] including 21 (8.7%) among women who initially reported not wanting another child [unwanted pregnancy rate 5.8 (95%CI: 3.6, 9.3) per 100 women-years].

**Conclusion:**

The persistence of relatively high unmet need among women receiving HIV care in a clinic with onsite FP services suggests the existence of barriers that must be identified and addressed.

## Introduction

Women of reproductive age account for about half of people living with the human immunodeficiency virus (HIV) globally [1]. Each year, some 1.5 million HIV-infected women become pregnant, mainly in sub-Saharan Africa [1]. HIV-infected women have higher needs for family planning (FP) for their own health and for preventing mother-to-child transmission of HIV. Unintended pregnancies are a major contributor to maternal mortality because they increase the risk of unsafe abortion, lead to frailty in women with high parity and closely spaced pregnancies, and cause obstructed labor in young women with premature pelvic development [2, 3]. A recent pooled analysis of data from six community-based studies in eastern and southern Africa with HIV serological surveillance and verbal-autopsy showed that, in HIV-infected women, the risk of death while pregnant or within 42 days of termination of pregnancy is eight times that of HIV-negative women. Likewise, an estimated 24% of deaths in all pregnant or post-partum women were attributable to HIV [4, 5]. The high risk of death persists even at higher CD4 count level [6]. In addition, over 90% of children infected with HIV acquire their infection from their mother during pregnancy, labor and delivery, or through breastfeeding [7]. Prevention of unintended pregnancies among women living with HIV is the second component of the World Health Organization's four-pronged approach to comprehensive prevention of mother-to-child transmission of HIV (PMTCT) [8, 9]. Provision of appropriate counselling and support, and contraceptives, to women living with HIV to meet their need for family planning and spacing of births has been shown to be a cost-effective intervention to prevent MTCT [10, 11]. In Uganda, for example, comparison of the effects of antiretroviral (ARV) prophylaxis (single dose nevirapine) for PMTCT to that of existing FP use shows that between 2008 and 2012, unwanted fertility would have accounted for 25% of pediatric infections while ARV-PMTCT would only have averted 18% [12]. Despite its potential impact on maternal and child health in the region, the use of contraceptives in sub-Sahara Africa is the lowest in the world. Recent estimates show that 60% of women in the region who want to avoid pregnancy have an unmet need for modern contraceptive methods (ie, currently are using no methods or a traditional method) [13]. Although there are no reliable data from the region quantifying the unmet need for modern contraceptive methods among HIV-infected women, studies suggest that HIV-infected women who know their status are substantially less likely to want more children compared to their HIV-negative peers [14–16] and the reported rate of unplanned pregnancies among HIV-infected women in the region remains high [16, 17]. Responding to the gap of unmet reproductive health needs of HIV-infected women, major international organizations including the United Nations have called for stronger linkages between reproductive health and HIV/AIDS care [18]. Understanding the fertility desires of HIV-infected women who know their HIV status, their contraceptive choices, and their pregnancy incidence is critical in meeting their reproductive health needs and preventing unwanted pregnancies. The aim of this study was to assess the fertility desires, the unmet need for FP, contraceptives choices, and the rate of pregnancies among HIV-infected women receiving care in a health center with onsite FP services in Kinshasa, the capital city of the Democratic Republic of Congo (DRC).

## Methods

**Study setting**: in November 2006, the University of North Carolina at Chapel Hill (UNC) in collaboration with the Kinshasa School of Public Health (KSPH), started providing family centered HIV care (including antiretroviral therapy) to HIV-infected women identified through a PMTCT Program at the Bomoi Health Center in Kinshasa. In June 2007, an onsite FP service managed by a nurse, was added to the program to provide family planning counseling and limited range of contraceptives (oral contraceptives, injectables (three-month DMPA), and condoms) free of charge to any HIV-infected woman or family member in the program, who self-expressed the need. All HIV-infected women who visited the clinic between November 2011 and May 2012, and agreed to be part of this study, were interviewed by a nurse using a structured questionnaire. The questionnaire included questions on obstetric history, sexual partner HIV status, history of contraceptive use, sexual behavior, fertility desires, and current use of contraceptive methods. Data from the interviews was entered in an Epi-info Database and linked with the electronic database containing information from routine follow-up visits of patients enrolled in the clinic. During routine follow-up visits in the clinic, pertinent information on HIV care and treatment were recorded. For women who reported a pregnancy or for whom a pregnancy was suspected on clinical grounds, the date of last menstrual period (LMP), the date of expected delivery, and the actual date of delivery or end of pregnancy were recorded.

### Measures and definitions

***Fertility desires***: during the interview, participants were asked if they ever wanted to have more children. The response options were “yes”, “no”, and “don't know”. Women who responded “no” were classified as not wanting more children and the remaining two responses were grouped together in one category to form a new binary variable. Participants were also asked if they have ever wanted to limit or space childbirth.

***Contraceptive use***: to assess the current use of effective contraceptives, participants were asked if they had sex since their last delivery and those who responded yes were asked if they were using condoms. If the answer was also affirmative, women were asked how often, and if they responded always, they were classified as consistent condom users. Irrespective of the answers to questions on condoms use, participants were also asked if they were using another contraceptive method beside condom. Those who responded yes were asked to specify which one. Participants who reported using any of the following: intra-uterine device (IUD), implant, injectable (three-month DMPA), oral contraceptive pills, or diaphragm/spermicides were classified as using a modern contraceptive method. Effective contraception was defined as using condom consistently or using a modern contraceptive method.

***Unmet need for family planning***: participants who responded not wanting to have more children ever, who were sexually active, and were not using effective contraception were considered to have unmet need for FP. Those not fitting this definition were considered not to have unmet need for FP. This analysis does not consider unmet need for spacing births, only unmet need for limiting births.

***Pregnancy Incidence***: participants started to accumulate follow-up time from the date of interview or, for women who were pregnant at the time of the interview, from the date of delivery, through their last visit to the clinic. The database was closed for this analysis on June 30, 2013. Women in care were not actively screened for pregnancy during routine visits. For any participant with a known pregnancy, key information related to the pregnancy was recorded in the routine follow-up database. Using those data, any pregnant woman whose date of LMP was after the date of interview or who gave birth at least 40 weeks after the interview date was considered to have had a new or incident pregnancy. Those who gave birth less than 40 weeks after their interview were considered to have been pregnant at the time of the interview (prevalent pregnancy).

**Socio-demographic variables**: other variables considered in the analysis include: age (in years) at the time of the interview, marital status dichotomized as married or cohabiting vs other; partner HIV status as reported by participants (infected, not infected, unknown), disclosure of one's HIV status to the partner (yes, no), number of children alive at the time of interview, and time in care since enrollment (time between enrollment and the interview).

**Statistical analysis**: the reported prevalence of contraceptive use and unmet need for family planning were calculated. Association of socio-demographic characteristics with unmet need for family planning was assessed using Chi Square for categorical variables and Wilcoxon Rank Sum test for continuous variables. Simple and multivariate logistic regression models were used to estimate the crude and adjusted odds ratios (OR) and 95% confidence interval (95%CI) for association of each characteristic considered with unmet FP needs. Pregnancy rates were calculated as the number of new pregnancies divided by follow-up time. Poisson regression was used to estimate the rate ratios (RR) and 95%CI comparing the rate of new pregnancies among women with unmet need and the rate of those without unmet need, adjusting for socio-demographic characteristics. All analyses were performed using SAS 9.3 (NC, Cary) and all tests were performed at 0.05 significance level. The study was approved by the UNC IRB and the KSPH ethical review committee.

## Results

A total of 699 HIV-infected women were interviewed for this study. At the time of interview, these women had been in care at the clinic for a median of 23.1 (interquartile range (IQR): 7.3, 35.3) months. The vast majority (80.6%) of them had received their HIV diagnosis through the PMTCT program. The median age at time of interview was 33 (IQR: 29, 37)years; 499 (71.7%) were married or cohabiting; 681 (97.4%) had been pregnant before. The median number of children alive was 2 (IQR: 1, 3) and 251 (35.9%) women reported 3 or more children (Table 1). Of all participants, 357 (62.9%) reported having shared their HIV status with their partner and 317 (55.8%) knew the HIV status of their partners, including 143 (25.2%) who reported their partner to be HIV-infected.

**Table 1:** Characteristics of 699 HIV-infected women interviewed between November 2011 and May 2012 in an HIV clinic in Kinshasa.

Characteristics	Number (%)
**Age in years:** median (IQR) in years	33 (29, 37)
**Marital status**	
Married or cohabiting	499 (71.70)
Single/divorced/widow/separated	197 (28.30)
**Currently pregnant**	
Yes	100 (14.31)
No	599 (85.69)
**Previous pregnancy(ies)**	
Yes	681 (97.42)
No	18 (2.58)
**Number of children alive**	
0	58 (8.30)
1	261 (37.34)
2	129 (18.45)
3 +	251 (35.91)
**Currently has a partner**	
Yes	568 (81.26)
No	131 (18.74)
**Partner HIV status**	
Infected	143 (25.18)
Not infected	174 (30.63)
Don't know	251 (44.19)
**Shared HIV status with partner**	
Yes	211 (37.15)
No	357 (62.85)
**Time since enrollment: median (IQR) in month** a	23.05 (7.31, 35.28)
**Ever wants more children**	
Yes/don't know	449 (64.33)
No	249 (35.67)
Use an effective contraceptive method*	
Consistent condom	96 (19.24)
Injectable (three-month DMPA)	104 (20.84)
Implant	1 (0.20)
Diaphragm/spermicide	1 (0.20)
Oral contraceptive pills	1 (0.20)
Dual method b	26 (5.21)
**Unmet contraceptive need** c, *	
Yes	88 (17.64)
No	411 (82.36)

IQR = interquartile range.

a Time between the date of enrollment at the clinic and the interview.

b Reported to always use a condom and to be using either an IUD, an implant, injectables, oral contraceptive pills, or diaphragm/spermicides.

c Women who reported no wanting any more children ever, who were sexually active and were not using an effective contraceptive method were classified as having unmet need for FP.

* The total is limited to 499 women who were sexually active at the time of interview

**Fertility desires and contraceptives use**: overall, 249 (35.7%) who reported not wanting more children ever. Of the 499 who reported to be sexually active at the time of interview, 253 (50.8%) reported to be using condoms, including 96 (19.2%) who reported to always use one. In addition, of 107 (21.4%) who reported using a modern method, almost all (104; 20.8%) used injectables, while diaphragm/spermicides, implant, and oral contraceptive pills were reported by a single women for each. Overall 177 (35.5%) women who were sexually active (at risk of becoming pregnant) were using an effective contraceptive method, including 26 (5.2%) dual method users who always used a condom and used another modern method (Table 1).

**Unmet need for family planning:** among the 249 women who did not want more children, 164 (65.9%) were sexually active. Of these, 100 (61.0%) reported using condoms; 40 (24.4%) used condoms consistently. In addition, 50 (30.5%) women reported using a modern contraceptive method (all injectable) including 14 (8.5%) dual method users. A majority of sexually active women who did not want more children (53.7%; 88 women) had unmet need for family planning. The overall prevalence of unmet need was 17.6% among participants who were sexually active at the time of interview.

**Characteristics of women with unmet need for family planning**: Compared to those without unmet need, in bivariate analyses, women with unmet need for family planning were more likely to be older (median age 36.0 vs 32.0 years, P-value <0.01) and to have more children (median number of children 3.0 vs 2.0, P-value <0.01). The length of the time since enrollment into care at the clinic, marital status, HIV status of the partner, and disclosure of one HIV's status to the partner were not statistically associated with unmet need for family planning (Table 2). In multivariate logistic regression, older age and higher number of children remain statistically associated with unmet need: adjusted OR 2.25 (95%CI: 1.24, 4.05) for every 10 years increase in age and 1.33 (95%CI: 1.14, 1.55) for every additional child respectively (Table 2). Restriction of the analysis to sexually active women did not change the results substantially except for revealing a trend toward less unmet need with increasing time in care. The adjusted OR associated with each year in the clinic before the interview was 0.83 (95%CI: 0.68, 1.02).**Incidence of pregnancies and characteristics of women with unwanted pregnancies**

**Table 2:** Unmet contraceptive needs and socio-demographic factors among 699 HIV-infected women interviewed between November 2011 and May 2012 in an HIV clinic in Kinshasa

	Unmet need a		Odds ratio	
	Yes	No	Crude (95%CI)	adjusted (95%CI)
**Age in years:** median (IQR) in years	36 (32, 39)	32 (28, 36)	3.00 (1.94, 4.63)	2.25 (1.24, 4.05)
**Number of live children: n (%)**	3 (2, 4)	2 (1, 3)	1.38 (1.23, 1.55)	1.33 (1.14, 1.55)
**Marital status: n (%)**				
Single/divorced/widow/separated	23 (11.68)	174 (88.32)	1	1
Married or cohabiting	65 (13.03)	434 (86.97)	1.13 (0.68, 1.88)	1.07 (0.44, 2.59)
**Partner's HIV status:** ***n (%)***				
Not infected	17 (9.77)	157 (90.23)	1	1
Infected	19 (13.29)	124 (86.71)	1.42 (0.71, 2.84)	1.26 (0.60, 2.64)
Don't know	36 (14.34)	215 (85.66)	1.55 (0.84, 2.85)	1.25 (0.58, 2.73)
**Shared status with partner: n (%)**				
No	32 (15.17)	179 (84.83)	1	1
Yes	40 (11.20)	317 (88.80)	0.71 (0.43, 1.16)	0.59 (0.29, 1.23)
**Time since enrollment: median (IQR) in months** b	26.13 (10.02, 39.77)	22.66 (7.11, 35.10)	1.01 (1.00, 1.02)	0.99 (0.98, 1.01)

IQR = interquartile range.

a Women who reported no wanting any more children, who were sexually active and were not using a modern contraceptive (reported not to always use a condom or not to be using either an IUD, an implant, injectables, oral contraceptive pills, or diaphragm/spermicides).

b Time between the date of enrollment at the clinic and the interview.

After the interview, 682 women had at least one follow-up visit by the time the database was closed for this analysis. During the median follow-up time of 22.2 (IQR: 20.2, 23.6) months, 96 (14.1%) women had a recognized pregnancy for an overall rate of 9.3 (95%CI: 7.6, 11.4) per 100 women-years. Of the 242 women with follow-up time who reported not wanting more children, 21 (8.7%) became pregnant during follow-up: a rate of unwanted pregnancy of 5.8 (95%CI: 3.6, 9.3) per 100 women-years (Table 3).

**Table 3:** Incidence of unwanted pregnancies and socio-demographic factors among 682 HIV-infected women interviewed between November 2011 and May 2012 in an HIV clinic in Kinshasa with at least one follow-up visit

		Incidence rate (95% CI)/ 100 women-years	Rate Ratio	
Characteristics	Person-months (cases)^a^		Crude (95%CI)	Adjusted (95%CI)
**Age in years:** median (IQR) in	390.78 (21)	5.78 (3.59, 9.29)	0.34 (0.16, 0.71)	0.29 (0.10, 0.85)
**Number of live children: n (%)**	390.78 (21)	5.78 (3.59, 9.29)	0.87 (0.68, 1.11)	1.02 (0.72, 1.45)
**Marital status: n (%)**				
Married or cohabiting	270.23 (14)	5.18 (3.07, 8.75)	1	1
Single/divorced/widow/separated	118.63 (7)	5.90 (2.81, 12.38)	1.14 (0.46, 2.82)	1.73 (0.44, 6.82)
**Partner's HIV status:** ***n (%)***				
Not infected	79.04 (6)	7.59 (3.41, 16.90)	1	1
Infected	101.11 (3)	2.97 (0.96, 9.20)	0.39 (0.10, 1.56)	0.38 (0.09, 1.57)
Don't know	114.12 (8)	7.01 (3.51, 14.02)	0.92 (0.32, 2.66)	0.85 (0.23, 3.14)
**SharedHIV status with partner: n (%)**				
Yes	95 (10)	5.02 (2.70, 9.33)	1	1
No	199.26 (7)	7.37 (3.51, 15.46)	1.47 (0.56, 3.86)	1.08 (0.29, 4.09)
**Time since enrollment: median (IQR) in months**^a^	390.78 (21)	5.78 (3.59, 9.29)	1.00 (0.98, 1.03)	1.02 (0.99, 1.05)
**Modern contraceptive method** b				
Yes	140.9 (7)	4.97 (2.37, 10.42)	1	1
No	249.88 (14)	5.60 (3.32, 9.46)	1.13 (0.46, 2.79)	1.31 (0.43, 4.00)

IQR = interquartile range.

a Time between the date of enrollment at the clinic and the interview.

b Reported to always use a condom or to be using either an IUD, an implant, injectables, oral contraceptive pills, or diaphragm/spermicides.

In both bivariate and multivariate analyses, younger age was the only factor that was statistically associated with unwanted pregnancies; for every 10 years increase in age, the adjusted rate ratio (RR) of having an unwanted pregnancy was 0.29 (95%CI 0.10, 0.85). Women who were not using an effective contraceptive method and those who were single/divorced/widowed or separated at the time of interview tended to have a higher rate of unwanted pregnancies: RR 1.31 (0.43, 4.00) and 1.73 (0.44, 6.82), respectively, though the rate ratio did not achieve statistical significance. Compared to women who reported their partner's HIV status as negative, those whose partner was HIV-infected also tended to experience fewer unwanted pregnancies: 0.38 (0.09, 1.57), but this was also not statistically significant (Table 3).

## Discussion

With a median of over 23 months in care, in a clinic with FP services onsite, just over 35% of HIV-infected women in our study who were sexually active were using an effective contraceptive method. Interest limiting childbearing was high: over one third reported not wanting any more children. Among women who did not want more children and were sexually active, 53% were not using any effective FP method. Those who did not want more children and were using effective contraception used condoms and/or injectable contraception. We know the women in this study were fertile, given that more than 80% were brought into care as part of PMTCT services. Overall, there was a relatively high incidence of pregnancy: 9.3 per 100 woman-years, including among women who did not want more children: 5.8 per 100 woman-years. Older age was associated with higher unmet need though younger women were more likely to experience unwanted pregnancy. In DRC, use of modern contraceptives is generally low among married women who are sexually active. In 2010, the estimated contraceptive use prevalence was 5.7% [19], substantially lower than in our study population. The prevalence of use of effective contraceptive methods other than condoms in our study (21%) was also substantially higher than what was reported in a similar population of HIV-infected women in care in neighboring Uganda (15%) [20] and the unmet need were also lower than the 28-35% reported among HIV-infected women in Nigeria [21]. This higher use of modern FP methods in our study was driven mainly by the use of injectables, which was provided free of charge in the clinic. Our findings provide additional evidence to the small but growing body of evidence suggesting that integrated FP and HIV services lead to increased use of modern FP methods among HIV-infected women [22–25]. However, despite the relatively increased utilization of effective contraceptive methods, a high proportion (53%) of participants who were sexually active and did not want more children were not using an effective method of contraception. The limited choices of contraceptive methods available at the clinic might explain some of this unmet need. The noted tendency of less unmet need among women with longer tenure in the clinic, though not statistically significant, suggests that an active educational campaign targeting HIV patients might help lower the rate of unmet need. In addition, active promotion of contraception by HIV-care providers, preferably during the same visits that women had for HIV care, might also have helped to reduce unmet need [26]. The drivers of unmet need likely go beyond information and access, however. HIV-infected women may find difficult to discuss their sexual activity and the need for contraception with providers, particularly if they are single, divorced, widowed, or separated. The barriers to accessing and using contraception that exist for non-HIV-infected women (e.g., partner disapproval of contraceptive utilization, negative provider attitudes, and fears about health risks and side effects from contraception) may be even more salient for HIV-infected women [27]. Further, insofar as religious leaders preach against contraception, religious beliefs may represent an additional barrier for HIV-infected women, because faith and religion were found to play a major role in how HIV-infected women cope with HIV in Kinshasa [28]. Overall, we suggest the need for further study to understand what motivates FP choices among HIV-infected women in our context.

In our study population, over 22% of new pregnancies were among women who had reported not ever wanting more children. Although even higher rates of unintended pregnancies have been reported among HIV-infected women in care in many settings [10, 20, 29, 30], it is important to note that our estimate doesn't account for pregnancies that might have ended in abortion which were not reported to clinicians. In addition to relatively high unmet need for family planning, we see evidence of method failure among women who were using an effective contraceptive method. A third of unwanted pregnancies occurred in women who were using injectables (and may have missed an injection before becoming pregnant) or condoms (and may have experienced a condom break). Women will benefit from increased access to methods less subject to failure, including long acting reversible contraception (IUDs, implants) and sterilization. Our study has a number of limitations. First, participants were interviewed only once and any changes in fertility intention or contraceptive use during the follow-up time are not known. For instance, some women who were using an effective method at the time of interview might have stopped during the follow-up time, leading to misclassification of their status as a contraceptive user. However, the resulting misclassification is likely to be non-differential with regard to incident pregnancies. Second, questions were not asked about why women chose or did not choose to use a particular method, limiting our ability to assess why women who said they did not want more children were not using an effective contraceptive method. Third, as described above, we only counted pregnancies that were known to health care providers, which means that our estimate of pregnancy rate is likely an underestimate. Finally, we assessed only unmet need for limiting births (i.e., women who didn't want any more children), not unmet need for spacing births (i.e., women who didn't want another child in the near future). This leads, however, to a conservative estimate of unmet need; the full unmet need for contraception is likely much higher if unmet need for spacing were to be included.

## Conclusion

In conclusion, our results add to the small but growing body of evidence suggesting that integration of FP services into HIV care leads to improved contraceptive use among HIV-infected women even in a setting with limited tradition of contraceptive use like DRC. However, the persistence of relatively high unmet need suggests other barriers beyond access that must be identified.
